# Rift Valley fever outbreak, Mauritania, 1998: seroepidemiologic, virologic, entomologic, and zoologic investigations.

**DOI:** 10.3201/eid0706.010627

**Published:** 2001

**Authors:** P Nabeth, Y Kane, M O Abdalahi, M Diallo, K Ndiaye, K Ba, F Schneegans, A A Sall, C Mathiot

**Affiliations:** Ministère de la Santé et des Affaires Sociales, Nouakchott, Mauritania.

## Abstract

A Rift Valley fever outbreak occurred in Mauritania in 1998. Seroepidemiologic and virologic investigation showed active circulation of the Rift Valley fever virus, with 13 strains isolated, and 16% (range 1.5%-38%) immunoglobulin (Ig) M-positivity in sera from 90 humans and 343 animals (sheep, goats, camels, cattle, and donkeys). One human case was fatal.


### Rift Valley Fever Outbreak, Mauritania, 1998: Seroepidemiologic, Virologic, Entomologic, and Zoologic Investigations

**Pierre Nabeth,* Yacouba Kane,† Mohameden O. Abdalahi,‡ Mawlouth Diallo,§ Kader Ndiaye,§ Khalilou Ba,¶ Fabien Schneegans,† Amadou Alpha Sall,§ and Christian Mathiot§**

*Ministère de la Santé et des Affaires Sociales, Nouakchott, Mauritania; †Centre National d'Elevage et Recherches Vétérinaires, Nouakchott, Mauritania; ‡Centre National d'Hygiène, Nouakchott, Mauritania; §Institut Pasteur de Dakar, Dakar, Senegal; and ¶Institut de la Recherche pour le développement, Dakar, Senegal

A Rift Valley fever outbreak occurred in Mauritania in 1998. Seroepidemiologic and virologic investigation showed active circulation of the *Rift Valley fever virus*, with 13 strains isolated, and 16% (range 1.5%-38%) immunoglobulin (Ig) M-positivity in sera from 90 humans and 343 animals (sheep, goats, camels, cattle, and donkeys). One human case was fatal.

In 1998, a Rift Valley fever outbreak was identified in Aioun El Atrouss, Hodh El Gharbi Region, Mauritania. This viral anthropozoonosis is transmitted by mosquitoes; it causes abortion in animals and illness ranging from febrile syndrome to hemorrhagic fever and death in humans (1). In 1987, following dam construction on the Senegal River, a major epidemic, with 200 human deaths, occurred for the first time in Mauritania (2). Since then, several smaller outbreaks have been reported, and regular circulation of the virus among cattle has been documented (3,4). We report laboratory and field investigations among animals and humans during the 1998 outbreak.

### The Outbreak

In September 1998, several patients with fever and hemorrhagic syndrome were admitted to the Hospital of Aioun El Atrouss, Hodh El Gharbi Region, Mauritania. Sera from two of these four patients were positive for *Rift Valley fever virus* (RVFV) by immunoglobulin (Ig) M detection by enzyme-linked immunosorbent assay (ELISA), virus isolation on cell cultures, and reverse transcription polymerase chain reaction, focusing on the S segment of the viral genome.

From October to the end of December, three epidemiologic investigations were undertaken in five localities in the Hodh El Gharbi region (Figure). The Hodh El Gharbi is an extensive livestock farming region in an arid area. In September 1998, rainfalls were exceptionally heavy, with a threefold increase over the 10-year average rainfall (86 mm vs. 26 mm).

During the investigations, serum samples were obtained from suspected cases in humans and animals (camels, goats, sheep, cattle, and donkeys) (Table). A suspected human RVFV case was defined as illness in any patient with fever (whether or not it was associated with hemorrhagic signs, icterus, or neurologic signs) occurring after September 1, 1998. Animal specimens were obtained from flocks in which abortions or stillbirths were reported in 1998. Based on a questionnaire among livestock breeders, the perinatal mortality rate (PMR) in flocks was estimated by using the ratio number of abortions, stillbirths, or deaths within 48 hours/number of females. Mosquitoes, sandflies, and biting midges were caught in CDC light traps. Mosquito larvae were also collected. Arthropods were sorted, pooled either by species and sex (for mosquitoes) or in polyspecific batches (sandflies, biting midges) in the field, and stored in liquid nitrogen. In light of the report on the possible involvement of rodents in

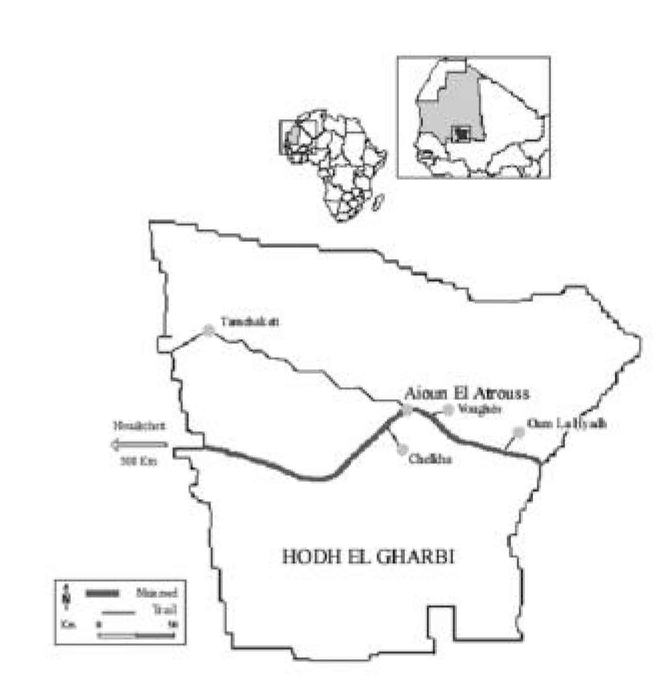

Figure. Map of Hodh el Gharbi region, Mauritania.

Address for correspondence: Amadou A. Sall, Institut Pasteur de Dakar, 36 Avenue Pasteur, BP 220, Dakar, Senegal; fax: 221-839-92-10; e-mail: sall@pasteur.sn

Table. Serologic, virologic, entomologic, and zoologic investigations, Rift Valley fever outbreak, Mauritania, 1998

**A. Seroepidemiologic and virologic results for humans and animals**

| | No. of samples | Recent infection | Past infection | Virus isolation |
|---|---|---|---|---|
| Humans | 90 | 16.7 (15/90) | 24.4% (22/90) | 2 |

| | | Flocks investigated | | | Animals tested | | | |
|---|---|---|---|---|---|---|---|---|
| Animal species | No. of females | NA[a] | PMR (%) | CI | Median age (years) | IgM isolation | IgG alone | Virus isolation |
| Sheep | 381 | 37 | 9.7 | 0, 20.7 | 1.5 | 34.8% (31/89) | 12.4% (11/89) | 6 |
| Goats | 471 | 223 | 47.4 | 33.3, 61.4 | 3.5 | 16.3% (23/141) | 24.8% (34/141) | 5 |
| Camels | 286 | 59 | 20.6 | 1.8, 39.4 | 7.5 | 2.6% (1/39) | 0% (0/39) | 0 |
| Cattle | 36 | 17 | 4.6 | 0, 9.3 | 2.5 | 1.5% (1.69) | 33.3% (23/69) | 0 |
| Donkeys | -- | -- | -- | -- | | 0% (0/5) | 20.0% (1/5) | 0 |

**B. Entomologic results**

| Mosquito species | No. | Abundance[b] | Virus isolation |
|---|---|---|---|
| *Anopheles pharoensis* | 8 | 1.5 | 0 |
| *Anopheles rhodesiensis* | 27 | 4.9 | 0 |
| *Anopheles rufipes* | 11 | 2.0 | 0 |
| *Culex antennatus* | 1 | 0.2 | 0 |
| *Culex decens* | 25 | 4.6 | 0 |
| *Culex neavei* | 68 | 12.5 | 0 |
| *Culex perfuscus* | 4 | 0.7 | 0 |
| *Culex poicilipes* | 191 | 34.9 | 0 |
| *Culex quinquefasciatus* | 211 | 38.6 | 0 |
| Total no. of mosquitoes | 546 | 100 | 0 |
| Sandflies | 524 | | 0 |
| Biting midges | 78 | | 0 |
| Total no. of arthropods | 1,148 | | 0 |

[a]NA = Number of abortions; PMR = perinatal mortality rate; CI = 95% confidence intervals.
[b]Abundance = number of individuals of one species/total number of mosquitoes collected.

the RVFV transmission cycle in South Africa (5), wild rodents were trapped concomitantly with the arthropods. Human and animal samples were tested for RVFV-specific IgG and IgM antibodies by ELISA as described previously (6) and for virus isolation in cell cultures and suckling mice. Viruses isolated were identified by indirect immunofluorescence with RVFV-specific monovalent hyperimmune mouse ascitic fluids, as well as complement fixation and seroneutralization tests (7). Arthropods were tested for the presence of RVFV by inoculating vector suspensions into cell culture, followed by identification as described for serum samples. Pooled rodent viscera were homogenized and inoculated intracerebrally into suckling mice for virus isolation.

Among the 90 human sera tested, 16.7% had evidence of recent (presence of IgM antibody or isolated virus) and 24.4% of past (presence of IgG antibody only) infection. Two virus strains were isolated. Among the 15 recently infected patients, median age was 26 years (range 10 to 45 years), male:female ratio was 2:0, and one death was reported. Proportions of hemorrhagic signs did not differ significantly among laboratory-positive and-negative cases (40.0% vs. 28.8%; p=0.5), suggesting that a cause other than RVFV should be considered to explain hemorrhages. Conversely, the prevalence of icterus and neurologic signs was significantly higher among positive cases (46.7% vs. 19.2%, p=0.04, and 53.3% vs. 20.5%, p=0.02, respectively), suggesting that these two signs were more specific indicators of RVFV in this human outbreak.

Among animals, 343 sera were tested from five species (sheep, goats, camels, cattle, and a donkey). Except for the donkey, all the species screened were positive for IgM antibodies, with prevalences ranging from 1.5% to 34.8%. These findings indicate not only widespread circulation of RVFV in the area but are also consistent with the high perinatal mortality observed in flocks, particularly among goats. The most affected species were sheep and goats, with an IgM prevalence of 34.8% and 16.3%, respectively; moreover, 11 RVFV strains were isolated from these two species (Table). However, the discrepancy between the perinatal mortality rates and the IgM-RVFV prevalence might suggest that other diseases causing abortion may have cocirculated in the area.

Among adult mosquitoes collected, *Culex* and *Anopheles* species were the most abundant. No RVFV strain was isolated, probably because the mosquito captures were undertaken at the end of the rainy season, when most breeding sites had dried up. This hypothesis is further strengthened by the small number of mosquitoes caught (546) and the absence of *Aedes* mosquitoes, a vector species for RVFV transmission in West Africa (8).

Seventy-three rodents belonging to five genera (*Gerbillus*, *Desmodilliscus*, *Acomys*, *Arvicanthis*, and *Jaculus*) and three families (*Gerbillidae*, *Muridae*, and *Dipodidae*) were captured in the same areas where mosquitoes were trapped, but no RVFV strains could be isolated and no serum was positive for IgM or IgG antibodies.

### Conclusions

An outbreak of Rift Valley fever occurred in the Hodh El Gharbi region of Mauritania in 1998. Because of the proportion of hemorrhagic signs in humans and the high rate of perinatal mortality among some animals, it cannot be ruled out that some other pathogen may have been involved in the outbreak; this hypothesis merits further investigation.

Since the 1987 epidemics, RVFV circulation among livestock has been documented in this region (3,4). However, outbreaks among humans and animals have probably been underreported, emphasizing the need to strengthen surveillance in the southern area of the country to prevent the potential spread of any epidemic focus to neighboring countries through nomadic animal husbandry.

Furthermore, the unique ecologic and environmental context makes the Hodh El Gharbi region of interest for research to further understand of factors influencing the emergence of this disease in West Africa.

### Acknowledgments

We thank R. Sylla, M. Ndiaye, M. Mondo, and L. Girault for their excellent technical assistance and Abdallaï Ould Valy and Mamoudou Diallo for their help during field investigation.

Dr. Nabeth is a medical epidemiologist in the department of statistics of the Ministry of Health in Mauritania. His areas of expertise and research interest include outbreak investigation and the epidemiology of infectious diseases.

### References

1. Peters CJ, Linthicum KJ. Rift Valley fever. In: Beran GW, Steele JH, editors. Handbook of zoonoses, Section B: Viral. 2nd ed. Boca Raton (FL): CRC Press; 1994. p. 125-38.
2. Digoutte JP, Peters CJ. General aspects of the 1987 Rift Valley fever epidemic in Mauritania. Res Virol 1989;140:27-30.
3. Zeller HG, Akakpo AJ, Ba MM. Rift Valley fever epizootic in small ruminants in Southern Mauritania (October 1995): risk of extensive outbreak. Ann Soc Belg Med Trop 1995;75:135-40.
4. Centre National d'Elevage et Recherches Vétérinaires. Activity report for 1997. Nouakchott, Mauritania: The Center; 1998.
5. Pretorius A, Oelofensen J, Smith S, van der Ryt E. Rift Valley fever virus. A seroepidemiologic study of small terrestrial vertebrates in South Africa. Am J Trop Med Hyg 1997;57:693-8.
6. Niklasson B, Peters CJ, Grandien M, Wood O. Detection of human immunoglobulin G and M antibodies to Rift valley fever virus by enzyme-linked immunosorbent assay. J Clin Microbiol 1984;19:225-9.
7. Digoutte JP, Calvo-Wilson MA, Mondo M, Traoré-Laminzana M, Adam F. Continuous cell lines and immune ascitic fluids pools in arbovirus detection. Res Virol 1992;143:417-22.
8. Fontenille D, Traore-Lamizana M, Diallo M, Thonnon J, Digoutte JP, Zeller HG. New vectors of Rift Valley fever in West Africa. Emerg Infect Dis 1998;4:289-93
